# How Do People Conceptualize Narcissism and Narcissistic Individuals?

**DOI:** 10.1111/jopy.70008

**Published:** 2025-07-11

**Authors:** Sarah Smith, Travis Proulx, Geoffrey Haddock

**Affiliations:** ^1^ School of Psychology Cardiff University Cardiff UK

**Keywords:** individual differences, lay perceptions, narcissism, thematic analysis

## Abstract

**Introduction:**

Although past decades have seen notable advances in the conceptualization and assessment of narcissism, scholarship examining lay conceptualizations of the construct remains limited.

**Method:**

We report two studies utilizing bottom‐up, participant‐driven methodologies to examine public understandings of narcissism and narcissistic individuals. In Study 1 (*n* = 202), we thematically analyzed layperson definitions of narcissism and compared their central contents with widely used narcissism measures. In Study 2 (*n* = 640), participants freely listed terms they associated with narcissistic or selfless acquaintances and rated them on a series of interpersonal dimensions (e.g., attributes, personal values).

**Results:**

Study 1 found that narcissism is most commonly conceptualized in relation to selfishness and vanity, and that divergences exist between public conceptualizations of narcissism and how it is operationalized in research. Study 2 found that although narcissistic acquaintances are ascribed greater grandiose relative to vulnerable traits (e.g., high extraversion, low agreeableness), they are also judged less favorably and perceived as placing greater (lesser) emphasis on self‐enhancement (self‐transcendence) values, relative to non‐narcissistic acquaintances.

**Conclusion:**

These findings broaden our knowledge of lay perspectives of narcissism and offer important theoretical (e.g., conceptualizations of narcissism) and practical implications (e.g., improving public communications regarding narcissism).

Since the days of the Roman poet Ovid, narcissism has captured the public imagination. In his mythological epic *Metamorphoses*, Ovid chronicles the tragic fable of Narcissus' vain self‐absorption. Beautiful and beloved, it was prophesied that Narcissus would live a long life if only he failed to recognize himself. Fatefully, after rejecting the advances of a river nymph, a parched Narcissus is lured to a pool of water, only to fall in love with his reflection. Paralyzed with self‐infatuation, he wastes away in solitude, leaving his only earthly trace—a burgeoning flower—his floral namesake.

Two millennia on from Ovid's tale there continues to be robust public interest in narcissism, with Google search interest in terms categorized under the topic of “narcissism” at their highest point in the UK since records began in 2004 (Google Trends, 2023). A breakout topic on TikTok, the hashtag #narcissist has over twelve billion views as of December 2023, ranking well above #ptsd (7.3 billion) and #ocd (6.8 billion). Content on the topic posted by social media influencers—such as “How to know if you're a narcissist” (Bartlett 2024)—is watched by millions and can impact how people think about narcissism. Indeed, countless social media posts show individuals discussing their own (and others') narcissism, proclaiming the importance of qualities such as selfishness (Lollie 2023) and vanity (Lopez 2023) in describing their own or others' narcissism. However, despite the widespread fascination with narcissism, relatively little is known about how lay persons conceptualize narcissism and narcissistic individuals. This is important—gaining a richer understanding of public perceptions of the construct could provide important conceptual insights and help to facilitate public understanding of scholarly work on narcissism. Accordingly, in this paper, we assess how people conceptualize narcissism and narcissistic individuals, and why these conceptualizations matter.

## 1 Psychological Perceptions of Narcissism and Narcissistic Individuals

Early conceptualizations of narcissism in the social/personality psychology literature can be traced to Ernest Jones's (1913/1951) description of individuals with a “God‐complex”. These individuals were construed as self‐admiring, self‐important, and exhibitionist, harboring fantasies of unlimited power and needing others' admiration. Over a century later, this constellation of traits described by Jones is remarkably similar to the personality facets captured by one of the most widely used measures of subclinical narcissism—the Narcissistic Personality Inventory (NPI; Raskin and Terry 1988).

Derived from the narcissistic personality disorder (NPD) DSM‐III diagnostic criteria, the NPI targets the prototypical “grandiose” narcissist, however, the literature now recognizes narcissism as comprised of, minimally, two separate dimensions—grandiose and vulnerable (see Miller et al. 2017). Grandiose narcissism is typified by a bold, outgoing, and dominant interpersonal orientation, while its vulnerable counterpart is characterized by hypersensitivity to rejection, self‐consciousness, and emotional fragility (Pincus et al. 2014; Rogoza et al. 2018). Indeed, these two sub‐forms differ markedly in their relationship to positive emotionality, with grandiose narcissism positively, and vulnerable narcissism negatively predicting greater levels of global self‐esteem (Rogoza et al. 2018; Weiss and Miller 2018).

Importantly, grandiose and vulnerable narcissism share a core sense of self‐importance, with individuals viewing themselves as deserving of special treatment (Miller et al. 2017). This ‘selfish core of narcissism’ (Campbell 2022) constitutes the binding principle of tridimensional models of narcissism. Within these models, grandiose and vulnerable narcissism are conceived of as two connected yet separate traits—bound by a foundational set of narcissistic features, typically labeled antagonism (Miller et al. 2016, 2017) or entitlement (Krizan and Herlache 2018). The trifurcated model of narcissism (Miller et al. 2016, 2017), for example, posits that the core component of narcissism is high antagonism (low agreeableness) manifested as low levels of trust, altruism and modesty (Miller et al. 2021). In the case of grandiose narcissism, this low agreeableness is combined with high levels of extraversion (e.g., assertiveness, drive, gregariousness); for vulnerable narcissism, it is mixed with high levels of neuroticism (e.g., vulnerability, self‐consciousness, shame).

## 2 Public Perceptions of Narcissism and Narcissistic Individuals

As noted earlier, social media is rife with posts from individuals willingly sharing stories of their self‐perceived narcissism, as well as detailed monologues expressing their own definitions of narcissism and the attributes/behaviors they associate with narcissistic individuals. Themes of selfishness, vanity, and exploitativeness (to name just three) are common. While such proclamations offer idiosyncratic perceptions of narcissism, there is some empirical research examining public perceptions of narcissism. Buss and Chiodo (1991) examined the acts that people considered prototypic of narcissism, with central themes including self‐centeredness, self‐absorption, and grandiosity. Park and Colvin (2014) found that while participants viewed their narcissistic companions as highly antagonistic, friends viewed narcissistic companions in relatively vulnerable terms, for example, having a critical and self‐defensive interpersonal style. Stanton et al. (2018) examined lay beliefs in narcissistic insecurity and found that grandiose narcissistic traits (e.g., arrogance) were viewed by the public as being linked to covert insecurity, emotional vulnerability, and jealousy of others. Miller, Gentile, et al. (2018) found that participants tend to view grandiose traits (e.g., low agreeableness and high extraversion) as more indicative of narcissism relative to vulnerable aspects (e.g., high neuroticism). Finally, Hyatt, Sleep, Lynam, et al. (2018) found that lay participants perceived grandiose and vulnerable narcissistic individuals as exhibiting anger under conditions of ego threat, with sadness being linked with vulnerable but not grandiose narcissism.

One common finding across such research is that narcissistic individuals are generally perceived negatively, though they can make positive first impressions that become more negative over time (Paulhus 1998). That said, narcissistic perceivers often evaluate narcissist targets more favorably (or, more specifically, less negatively) than non‐narcissistic perceivers—an effect known as *narcissistic tolerance* (Hart and Adams 2014). Indeed, narcissism is positively associated with evaluations of others' narcissistic behaviors (Burton et al. 2017) and ratings of hypothetical characters possessing narcissistic traits (Wallace et al. 2015).

However, thus far, relevant research assessing perceptions of narcissism and narcissistic individuals has utilized top‐down approaches where participants rate narcissistic targets along predetermined traits and social outcomes. To our knowledge, no research has adopted bottom‐up approaches whereby participants freely describe their understandings of narcissism. Elsewhere, research using a bottom‐up approach has demonstrated marked differences between academic and public conceptualizations of other fundamental psychological constructs, such as empathy and the Big Five traits (Hall et al. 2019, 2021). Such research has revealed several components of these constructs identified by participants that are absent from standard measurement scales, and vice versa. This is important because it implies that laypersons' conceptualizations of core psychological phenomena may not neatly map onto the primary aspects of the same phenomena as broadcast in the research literature or the original myth‐based conceptualization.

## 3 The Present Research

Our research addresses the following fundamental questions: How do people define narcissism? To what extent are lay definitions of narcissism captured in commonly used narcissism scales? How desirable is narcissism perceived to be, both at the concept and person level? What attributes (e.g., Big Five traits, personal values) are associated with narcissistic individuals? And finally, how do narcissistic and non‐narcissistic individuals evaluate narcissism and narcissistic individuals?

Study 1 focused on understandings of narcissism at the *concept* level. Here, participants freely described their own personal definition of narcissism and the extent to which they perceived one of the most widely used measures of the construct, the Narcissistic Personality Inventory (NPI; Raskin and Terry 1988) as representative of their own conceptualization. Study 2 examined perceptions of narcissism at the *person* level. Here, participants freely listed the characteristics they associated with a narcissistic (or selfless) acquaintance and rated this individual on a set of attributes. Across both studies, we measured participants' own level of narcissism and explored how these scores influence perceptions of the concept of narcissism and narcissistic acquaintances.

## 4 Study 1—How Do People Define Narcissism?

Study 1 adopted a bottom‐up approach, where participants provided their own definition of “narcissism”, listed the traits and behaviors that they associated with narcissism, and indicated how desirable they perceived these traits and behaviors to be. Participants also listed terms that they felt best represented the opposite of narcissism. Finally, we explored the extent to which participants endorsed the items of the NPI‐13 (Gentile et al. 2013; Raskin and Terry 1988) as reflective of their own personal conceptualization of narcissism. Importantly, we explored the relationships between participants' NPI scores and the content of their definitions, perceived valence of traits and behaviors exemplifying narcissism and NPI endorsement.

We predicted that narcissistic traits would generally be perceived unfavorably (i.e., significantly lower than the scale mid‐point), but that self‐reported narcissism would positively correlate with appraisals, in line with the narcissistic tolerance perspective.

## 5 Method

### 5.1 Participants

We recruited 212 UK participants via Prolific, who each received £1.24 for their participation. Six participants were excluded for failing an honesty check item (i.e., they used an additional source, such as a dictionary, when reporting their definition of narcissism). Four other participants were excluded for incorrectly responding to attention check items. This resulted in a final sample of 202 (96 males, 103 females, 1 other, 2 did not to say; 57% with a college degree; *M*_age_ = 38.01; SD_age_ = 14.95).

Our sample size was guided by affordability and extant research on lay perceptions of attributes (Hall et al. 2019, Study 1). A sensitivity power analysis conducted using G*Power indicated that our sample was sufficiently powered (power = 0.80, *α* = 0.05, two‐tailed) to detect correlations of |0.19| and higher.

### 5.2 Materials and Procedure

#### 5.2.1 Personal Definition of Narcissism

We collected data online via Qualtrics. First, participants provided their personal definition of the term narcissism by typing their definition into a text box. There were no time, character, or detail limits for this task, though they were asked to refrain from using a dictionary or thesaurus.

#### 5.2.2 Traits and Behaviors Associated With Narcissism

Following the definition task, participants listed five traits or behaviors that they personally associated with narcissism. Participants next evaluated each of their listed responses for valence (1 = extremely negative; 7 = extremely positive). For the present study's purposes, we were only interested in the valence ratings ascribed to each feature. However, the traits and behaviors were analyzed using prototype analysis for a separate study.

Participants also indicated their familiarity with the term ‘narcissism’, as well as how confident they felt in their own understanding of the term (0 = not at all familiar/confident, 100 = extremely familiar/confident).

#### 5.2.3 Opposite of Narcissism

Next, participants completed two short tasks designed to determine how well various terms represent the opposite of narcissism (which is relevant to Study 2). First, participants rated four words—selfless, altruistic, modest, and generous—as opposites of narcissism (1 = definitely not, 5 = definitely yes) and were also asked to list any other words that came to mind and rate them accordingly. Second, participants selected one word from that selection that they felt best represented the opposite of narcissism.

#### 5.2.4 Narcissistic Personality Inventory—Likert Version

Participants completed the Likert rating version of the NPI‐13 (Gentile et al. 2013; Raskin and Terry 1988). Within the measure we included an attention check item that required participants to select a certain number. Participants rated their agreement with the extent to which each statement applied to them personally, for example, “I find it easy to manipulate people” and “I like to show off my body”. We calculated average total NPI scores for each participant (*M* = 2.95; SD = 0.82; *α =* 0.83).

#### 5.2.5 Filler Measures

After completing the NPI, participants completed three filler measures. These included two single‐item measures of self‐esteem (Gebauer et al. 2008; Robins et al. 2001) and importance ratings of Schwartz's (1992) four value types (self‐transcendence, self‐enhancement, openness, and conservation). Data collected from these filler measures were not intended to be analyzed; rather, they were included to avoid participants completing the next task immediately after completing the NPI.

#### 5.2.6 Endorsement of NPI Items

Participants rated each of the NPI‐13 items (presented in a random order) for how well each item matched their own definition of narcissism (1 = not narcissistic, according to my definition, 9 = extremely narcissistic, according to my definition). All items were rephrased from self‐report to describe a range of feelings, beliefs, attitudes and behaviors. For example, the item “I expect a great deal from other people” was rephrased as “Expecting a great deal from other people”.

Finally, participants reported their gender, age, educational status, and political orientation.

### 5.3 Narrative Coding of Personal Definitions

Narrative coding of the definition data was a three‐part process. First, all definitions were allocated an accuracy score based classifications from Hall et al. (2019): (1) *Senseless, silly, not credible as an answer*, (2) *Seems to misunderstand what it means, or says they have no idea*, (3) *Somewhat suggests the trait* [e.g., *naming just one of several possible facets of the trait*], *and* (4) *Fits an obvious way of defining the trait*. For example, the definitions: “someone selfish and arrogant”, “rejection of other people's ideas”, “someone that dislikes something a lot”, and “self‐praise is donkey praise” received accuracy scores of 4, 3, 2, and 1, respectively.

Second, an inductive narrative thematic analysis process was used to sort the definitions into conceptually similar categories (see Hall et al. 2019). Each definition was then allocated a code that pertained to each of the major categories (e.g., code 1 = Social Selfishness). Definitions could be allocated multiple different codes; however, if participants made multiple statements that referred to only one code, that code was allocated only once. A full coding manual for the 10 Narrative Narcissism Codes is available within the Supporting Information (see Table S1).

Finally, we examined the extent to which participants' definitions of narcissism overlapped with the contents of common assessment measures: NPI‐40 (Ackerman et al.'s 2011, three‐factor solution; Ackerman et al.'s 2016, five‐factor solution; Raskin and Terry's 1988 seven‐factor solution), the Grandiose Narcissism Scale (GNS; Foster et al. 2015), and the Five Factor Narcissism Inventory (Glover et al. 2012). Definitions were given a facet code if they conceptually matched the relevant facet from any of the measures (definitions could be allocated more than one code). For example, the definition “narcissism is when a person holds superior beliefs about themselves” would receive the following facet codes: NPI‐7 Superiority, NPI‐5—Superiority, GNS—Superiority, and FFNI—Arrogance.

The first author conducted all coding. Following Syed and Nelson's (2015) guidelines, we randomly selected 20% of the definitions to be independently coded by a trained research assistant. The research assistant indicated agreement with the coding decisions of the first author 85% (narcissism narrative codes) and 90% (facet codes) of the time.

## 6 Results

We begin by describing participants' self‐reported knowledge about narcissism and the perceived desirability of narcissistic traits and behaviors, before highlighting the emergent themes that were present in participants' definitions. Next, we compare participants' definitions with the content of common narcissism measurement scales and examine participants' endorsement of the NPI as reflective of their own conceptualization of narcissism. Finally, we explore participants' chosen terms that best conceptualize the opposite of narcissism.

### 6.1 Self‐Reported Accuracy and Knowledge

Four definitions received accuracy ratings below 3 and were removed from subsequent analyses without impacting the overall pattern of findings. The remaining definitions received ratings of either 3 (18.3%) or 4 (79.7%). Familiarity and confidence scores regarding the term ‘narcissism’ were strongly correlated (*r*(200) = 0.82, *p* < 0.001), so we created a ‘knowledge’ index comprised of an average of participants' score on both variables. There were no associations between knowledge scores and NPI scores, age, or political orientation (all *p*s ≥ 0.068). We also found no gender differences in self‐reported knowledge (*M*_male_ = 67.64, *M*_female_ = 63.66; *t*(194.47) = 1.41, *p* = 0.162, Cohen's *d* = 0.20).

### 6.2 Perceived Desirability of Narcissistic Traits

To examine participants' perceived desirability of narcissistic traits and behaviors, we computed an average valence rating for each participant. Narcissistic attributes (*M* = 1.89, SD = 0.76) were evaluated significantly less positively than the scale midpoint, *t*(197) = −38.89, *p* < 0.001; Cohen's *d* = −2.76. Participant narcissism (i.e., total NPI score) was positively associated with perceived desirability of narcissism, (*r*(198) = 0.18, *p* = 0.011), such that narcissistic participants were less negative in their perceived desirability of narcissistic traits and behaviors. This pattern is consistent with the narcissistic tolerance hypothesis (Hart and Adams 2014). Neither age nor political orientation were related to valence scores (*p*s ≥ 0.252) and no gender difference was found, *t*(194) = 0.707, *p* = 0.582, Cohen's *d* = 0.10.

### 6.3 Narcissism Narrative Codes

We present the percentages of participant narratives receiving each Narrative Narcissism Code in Table 1. The mean number of codes allocated per participant was 2.14 (SD = 1.05; range 1–6). Social Selfishness (persistently prioritizing oneself above others; having a self‐centered worldview) was most frequently mentioned by participants (60%). Sample narratives that received this code were: “Being selfish and not caring about other people” and “…putting your own needs before everyone else”. Additionally, 41% of participants mentioned Vanity (excessive admiration of one's physical and mental attributes and abilities). Sample narratives that received this code were: “An unusually deep‐seated love for the self, including body image” and “Obsessed with oneself”.

**TABLE 1:** Percentages of participant definitions allocated each narcissism code (Study 1).

	Code name	Example definition	%
1	Social selfishness	“Someone who only thinks about themselves”	60
2	Vanity	“Being vain; loving yourself”	41
3	Impaired empathy	“Struggling to see from others' points of view”	29
4	Relational grandiosity	“Someone who feels they are superior to others”	27
5	Social aggression	“Gets enjoyment from putting others down”	21
6	Stubbornness	“Refuses to see flaws in their behavior”	9
7	Obliviousness	“Self‐obsessed but unaware”	9
8	Attention‐seeking	“Having the desire to be the center of attention”	8
9	Deservingness	“Narcissism is characterized by self‐entitlement”	5
10	Emotional fragility	“…it comes from a place of deep‐seated insecurity”	4

*Note:* Percentages exceed 100 as some participant definitions mentioned multiple codes. *N* = 198.

Furthermore, at least a quarter of participants included Impaired Empathy (diminished concern for others' thoughts, emotions, and opinions; 29%) and Relational Grandiosity (preoccupation with one's own specialness and superiority *over others*; 27%) in their definitions. In contrast, the codes Stubbornness (refusing to change one's view or position, or to admits one's faults), Obliviousness (having no self‐awareness over the impact of one's actions or how they are perceived by others), Attention‐Seeking (engaging in exhibitionist, self‐promoting behaviors), Deservingness (believing that you are innately entitled to others' attention, admiration and recognition), and Emotional Fragility (a tendency toward low or unstable self‐esteem) were mentioned by less than 10% of participants.

### 6.4 Correlates of Narcissism Narrative Code Allocation

Next, we examined relationships between participant narcissism and the allocation of individual codes. While total NPI score was unrelated to the allocation of any particular codes (all *p*s ≥ 0.020), it was negatively associated with the number of codes allocated (*r*_*s*_(196) = −0.21, *p* = 0.004), with participants scoring high in narcissism generating definitions with fewer codes. We also examined age, gender, and political orientation as correlates of code allocation. Age was correlated with Vanity code allocation (*r*_pb_(196) = 0.26, *p* < 0.001), suggesting that older participants may consider vanity to be a more salient aspect of narcissism. Political orientation and gender were both unrelated to code allocation (all *p*s ≥ 0.032).

### 6.5 Comparing Participant Definitions With Common Measurement Content

Table 2 shows the top three facets from each scale that were most frequently mentioned in participants' definitions (see Table S2 for percentages for all facet codes with example excerpts). The mean number of facet codes allocated per participant was 4.86 (SD = 3.26; range 0–22). The facet code “FFNI—Entitlement” was the most commonly allocated, with 51% of definitions demonstrating conceptually similar content to this facet. The second most allocated facets were “FFNI—Arrogance” and the NPI‐7 and NPI‐5 Superiority facets (all 44%).

**TABLE 2:** Top and bottom three facets of each narcissism measure allocated to definitions (Study 1).

Measure	Top facets (%)	Bottom facets (%)
FFNI	Entitlement (51)	Thrill‐Seeking (0)
	Arrogance (44)	Grandiose Fantasies (0)
	Lack of Empathy (32)	Acclaim‐Seeking (0)
GNS	Superiority (44)	Self‐Sufficiency (0)
	Exploitativeness (23)	Authority (4)
	Exhibitionism (10)	Vanity (6)
NPI‐7	Superiority (44)	Self‐Sufficiency (2)
	Exploitativeness (17)	Authority (4)
	Exhibitionism (11)	Vanity (6)
NPI‐5	Superiority (44)	Leadership (5)
	Manipulativeness (16)	Vanity (7)
	Exhibitionism (10)	Exhibitionism (10)
NPI‐3	E/E (25)	L/A (5)
	GE (15)	GE (15)
	L/A (5)	E/E (25)

*Note:* Percentages exceed 100 as participant definitions could mention multiple codes.

Abbreviations: E/E, entitlement/exploitativeness; FFNI, five factor narcissism inventory; GE, grandiose exhibitionism; GNS, Grandiose Narcissism Scale; LA, leadership/authority; NPI‐7, NPI‐5, NPI‐3, Narcissistic Personality Inventory seven‐, five‐, and three‐factor solutions, respectively.

Regarding the facets that were least reflected in participants' definitions, no definitions received the FFNI Acclaim‐Seeking, Grandiose Fantasies, or Thrill‐Seeking facet codes. Additionally, very few definitions received facet codes relating to the leadership/authority dimensions of the construct. This suggests that public understandings of narcissistic individuals as authoritative or risk‐taking are less salient to most participants than notions of narcissistic individuals' arrogance and self‐entitlement.

### 6.6 Endorsement of the NPI‐13

Participants rated each NPI‐13 item for how well it reflected their own personal definition of narcissism. The mean score across all items was 6.44 (SD = 1.39). The item receiving the highest rating was: “Finding it easy to manipulate people” (*M* = 7.24; SD = 1.93), and the lowest rating item was: “Feeling as though you are a good person because everybody keeps telling you so” (*M* = 4.63; SD = 2.24).

Next, we conducted a repeated measures ANOVA to compare levels of endorsement across facets. There was a significant effect of facet type, *F*(1.70, 334.07) = 27.79, *p* < 0.001, *η*_*p*_^2^ = 0.097. Pairwise comparisons revealed that the NPI‐EE facet (*M* = 6.72; SD = 1.51) was perceived as more representative of narcissism compared to the NPI‐GE facet (*M* = 6.08; SD = 1.72; *p* < 0.001; Cohen's *d* = 0.39). No differences were found between scores on the NPI‐LA facet (*M* = 6.61; SD = 1.65) and the NPI‐EE facet (*p* = 0.589); however, the NPI‐LA facet was seen as more representative than the NPI‐GE facet (*p* < 0.001, Cohen's *d* = 0.33).

### 6.7 Opposite of Narcissism

We conducted a repeated measures ANOVA to examine which term (selfless, altruistic, generous, modest) was perceived as best representing the opposite of narcissism. There was a significant effect, *F*(2.85, 558.83) = 17.01, *p* < 0.001, *η*_*p*_^2^ = 0.080. Post hoc pairwise comparisons revealed that ‘selfless’ (*M* = 4.26; SD = 1.24) and “modest” (*M* = 4.10; SD = 1.19), were seen as significantly more representative of the opposite of narcissism than ‘generous’ (*M* = 3.82; SD = 1.10), and ‘altruistic’ (*M* = 3.74; SD = 1.19; all *p*s < 0.001; Cohen's *d*s 0.24–0.45), with no difference in ratings between “selfless” and “modest” (*p* = 0.231).

To assess what other words might be representative of the opposite of narcissism, we conducted a frequency analysis on all words offered by participants. Of the 112 unique words generated, those listed by 10 or more were: Kind (28), Empathetic (21), Caring (15), Humble (12), and Considerate (12). Participants who offered additional terms (*n* = 128) still rated the term ‘Selfless’ (*M* = 4.29; SD = 1.21) as more representative of the opposite of narcissism than their suggested alternatives (*M* = 3.98; SD = 1.05; *t*(127) = 3.18, *p* < 0.001, Cohen's *d* = 0.28). Furthermore, there was a significant difference between the terms participants selected as best representing the opposite of narcissism (*X* ^2^(4) = 159.53, *p* < 0.001), with 55% of respondents selecting selfless, and less than a quarter selecting modest (16%), altruistic (13%), generous (4%), or “other” (12%).

## 7 Discussion

Using a bottom‐up approach, Study 1 revealed that the most commonly mentioned aspect of narcissism was social selfishness, followed by vanity, relational grandiosity, and impaired empathy. That this constellation of traits was most prominent in the minds of laypeople converges with research demonstrating that people tend to view grandiose (vs. vulnerable) aspects of narcissism as more prototypical (e.g., Carlson et al. 2012; Miller, Gentile, et al. 2018). Furthermore, that social selfishness was the trait most frequently mentioned by participants suggests some level of consensus between the public views of narcissism and contemporary models of the construct that propose the underlying narcissistic nucleus to be antagonism/entitlement (Campbell 2022; Miller et al. 2021).

That said, our findings also signaled areas of non‐overlap between lay conceptualizations of narcissism and the content of widely used narcissism scales. For example, although the majority of participants' narcissism definitions focused on social selfishness, items *explicitly* capturing this aspect across the measures we examined were scarce. Indeed, only the FFNI and GNS contain items that *directly* tap into this theme (e.g., “I sacrifice my own needs for those of others” [FFNI‐Entitlement] and “I deserve more out of life than other people” [GNS—Entitlement]). Moreover, more than two‐fifths of the participants emphasized vanity in their definitions of narcissism. Yet, of the 40 items that comprise the NPI, only three directly relate to narcissistic vanity (e.g., “I like to show off my body”), with the FFNI completely lacking in items directly denoting vanity.

Likewise, regarding the theme of relational grandiosity—another prominent aspect of participants' narcissism concepts—only the FFNI and GNS specifically address the comparative nature of this feature (i.e., feeling special *in comparison to* others) with items such as “I only associate with people of my caliber” (FFNI‐Arrogance) and “I'm more talented than most other people” (GNS—Superiority). Conversely, the NPI (ratings version) measures superiority via items such as “I will be a success” and “I like to be complimented”, which may not necessarily reflect the relational element of narcissistic grandiosity emphasized by our participants.

In addition to the scarcity of items directly capturing key aspects of lay definitions, we also found that many scale items captured phenomena absent from participants' concepts. For example, participants scantly mentioned leadership/authoritative tendencies in their personal definitions, yet the Leadership/Authority facets of the NPI‐40 include the largest proportion of items relative to all other facets. It should be noted, however, that previous research has found both lay raters and professionals with expertise in these constructs (e.g., clinicians, academicians) rate traits such as assertiveness and ambitions as prototypical of narcissism (Miller, Gentile, et al. 2018). Nonetheless, these aspects were not *spontaneously emphasized* by our participants. Additionally, three facets of the FFNI (Thrill‐Seeking, Acclaim‐Seeking, and Grandiose Fantasies), as well as the GNS Self‐Sufficiency facet, were found to share minimal, if any, conceptual similarity with lay definitions, suggesting that these aspects are not readily salient in public conceptualizations of narcissism.

Further, participant narcissism was positively associated with the perceived desirability of narcissistic traits. These findings support and extend the narcissistic tolerance hypothesis. Whereas past research has found that narcissistic (vs. non‐narcissistic) individuals perceive hypothetical characters possessing narcissistic traits as more likable (Burton et al. 2017; Hart and Adams 2014), the present study demonstrates that the same effect is replicated when using a bottom‐up, concept‐level approach.

Study 1 also provided further knowledge regarding the relationship between conceptualizations of narcissism and demographic variables. While gender was found to be unrelated, age represented a significant factor in one's concept of narcissism, such that older participants were more likely to emphasize vanity when defining the construct and also more greatly endorsed the NPI as representative of their own narcissism concept. This indicates potential generational differences in public perceptions of narcissism; indeed, age represents a negative predictor of vanity (Wetzel et al. 2020). Consequently, younger participants may be more likely to normalize the trait, which accordingly becomes less readily salient in their narcissism conceptualizations.

Overall, Study 1 provides initial evidence that lay conceptualizations of narcissism tend to emphasize its social selfishness and vanity aspects. However, while Study 1 represents a novel, bottom‐up approach to public understandings of the *concept* of narcissism, it did not address how people conceptualize narcissism at the *person* level. Thus, Study 2 focuses on perceptions of narcissistic individuals and the personal values, personality traits, and interpersonal qualities that people associate with narcissistic individuals.

## 8 Study 2—How Do People Perceive Narcissistic (vs. Selfless) People?

While Study 1 analyzed personal definitions of narcissism, Study 2 explores a different question—how do people conceptualize narcissism at the *person* level? Specifically, we asked the following questions: what freely listed words do people generate when asked to describe a narcissistic (vs. selfless) acquaintance? How desirable do people perceive these words to be? How do people evaluate narcissistic (vs. selfless) targets across a range of attributes, such as their personal values, Big Five, and interpersonal traits [warmth, competence, liking, and success]? And, finally, to what extent might one's own narcissism influence evaluations of narcissistic (vs. selfless) acquaintances?

We randomly assigned participants to think about an individual they knew that they would consider to be either very narcissistic or very selfless (the label most perceived as the opposite of narcissism, from Study 1). We asked participants to list five words that they associated with that individual's character, and how desirable they rated their chosen terms to be before indicating their perceptions of that individual's attributes and personal values.

Based on extant theoretical frameworks and research, we focused on perceptions of a set of outcomes: personal values, interpersonal traits [warmth, competence, liking and success], Big Five traits, self‐esteem, and political orientation. First, *values* represent trans‐situational goals and ideals that serve as guiding principles in an individual's life (Schwartz et al. 2012). Research has positively linked all facets of narcissism to self‐enhancement values (e.g., wealth, ambition, personal success), and antagonistic, neurotic, and communal narcissism negatively to self‐transcendent values (e.g., equality, honesty; see Nowak et al. 2022). Additionally, agentic narcissism (the sub‐facet of grandiose narcissism typified by self‐enhancement in the agentic domain [e.g., ambition, drive; see Sedikides 2021]) has been found to be positively correlated with openness to change values (e.g., freedom, curiosity, adventurousness) and negatively correlated with conservation values (e.g., politeness, respect for tradition, obedience). However, to the best of our knowledge, research has not yet examined the types of values people *perceive* to be important for narcissistic individuals.

Second, regarding perceptions of *interpersonal traits*, we focused on warmth, competence, liking, and success. Although NPI scores have been significantly related to perceptions of greater agency (e.g., being seen as having high aspirations and being productive), but not communion (e.g., being seen as sympathetic, considerate, and giving; see Park and Colvin 2014), to our knowledge, research is yet to explicitly assess perceptions of narcissistic individuals' warmth and competence. This is important because, as per the Stereotype Content Model (SCM; for a review see Fiske 2018), warmth and competence represent fundamental aspects of social perception. Additionally, we focused on perceptions of liking, as previous research has found that perceiver narcissism predicts perceived tolerance for others' narcissism. For example, narcissism was positively associated with liking fictional characters described as possessing narcissistic traits (Hart and Adams 2014). Lastly, we focused on perceptions of success, as research has positively associated grandiose narcissism with greater academic and occupational success throughout one's life (O'Reilly and Pfeffer 2021).

Third, regarding the Big 5, research has linked narcissism positively to perceived extraversion and negatively to perceived agreeableness, as rated by close others (Carlson et al. 2012). However, the literature on other perceptions of narcissism has heavily relied on the use of procedures where participants are asked to recruit friends to serve as raters. Research examining the relationships between participant raters and their targets found that participants tend to nominate raters who like them and are more likely to describe the target's personality in positive ways (Leising et al. 2010).

Finally, at an exploratory level, we examined perceptions of *self‐esteem* and *political orientation*. Although self‐esteem and grandiose narcissism are phenotypically distinct, they are positively correlated (Hyatt, Sleep, Lamkin, et al. 2018). Yet, research has demonstrated that people generally hold the belief that narcissism is linked to covert insecurity (Stanton et al. 2018). Regarding political orientation, perceptions of others' political ideologies predict important social outcomes (Westwood et al. 2018). While perceptions of narcissistic individuals' political orientations are yet to be empirically examined, people on both sides of the political spectrum have been found to be equally narcissistic (Hatemi and Fazekas 2018).

A number of predictions were made. First, to the extent that narcissistic individuals attach greater importance to self‐enhancement values and lower importance to self‐transcendence values relative to non‐narcissistic individuals, we predict that narcissist acquaintances will be perceived as attaching greater importance to self‐enhancement values and lower to self‐transcendence values compared to selfless acquaintances. Further, given our previous findings regarding evaluations of narcissism, we expect narcissistic acquaintances to be evaluated more negatively than selfless acquaintances. We made no predictions on perceived self‐esteem and political orientation. Finally, based on the narcissistic tolerance hypothesis (Hart and Adams 2014) and the results of Study 1, we expected that evaluations of narcissistic acquaintances would be positively correlated with participants' own narcissism.

## 9 Method

### 9.1 Participants

We recruited 682 UK participants, via Prolific (who each received 94p for their participation) and a University participant panel (who each received course credit). A mixed sample was guided by affordability. Participants from Study 1 were unable to participate in Study 2. Forty‐two respondents were excluded for failing the honesty check or attention check; the final sample was 640 (317 from Prolific; 323 from university panel; 448 females, 181 males, 9 other, 2 did not to say; 29% with a college degree; *M*_age_ = 29.38; SD = 14.45).

Based on guidelines by Sommet et al. (2023) for detecting patterns of moderated regression effects (in our case, a mixed design with a predicted significant simple slope in one condition), a sample size of 624 was required to achieve 80% power at *α* = 0.05. Further, a post hoc sensitivity analysis using G*Power (Faul et al. 2009) confirmed that our final sample (*N* = 640) was sufficient to detect an interaction effect of *f* ^2^ = 0.012.

### 9.2 Materials and Procedure

#### 9.2.1 Word Generation Task

Materials were presented via Qualtrics. Participants were randomly assigned to consider someone that they knew to be either very *narcissistic* or very *selfless*. In both cases, participants were asked to name their chosen acquaintance and list five words that they would use to describe that person's character. They were requested to limit their responses to single words or two‐word phrases and to resist using tools such as an online dictionary or thesaurus.

Following this, participants completed an honesty check (i.e., asking them to confirm whether they generated all words themselves) before evaluating each of their listed traits for valence (1 = extremely negative; 7 = extremely positive).

#### 9.2.2 Perceptions of Targets' Values

Next, participants completed a series of tasks regarding their named acquaintance. First, to measure perceived values, participants completed a shortened version of the Schwartz's Value Survey (SVS; Schwartz 1992). Participants rated the extent to which they perceived Schwartz's four primary value types (self‐transcendence, self‐enhancement, openness, and conservation) as important to their named acquaintance (0 = not at all, 100 = a great deal). Four values were used for each main value type (e.g., perceived self‐transcendence was measured with the following item: “Please rate how important you feel honesty, equality, forgiveness, and protecting the environment is to [named person]”).

#### 9.2.3 Perceptions of Targets' Personality Traits

Participants evaluated their target's Big 5 personality traits using the Ten‐Item Personality Inventory (TIPI), a measure with acceptable psychometric properties (Gosling et al. 2003). Participants rated their target on various traits (e.g., extraverted, enthusiastic; 0 = not at all, 100 = a great deal). Additionally, participants rated their target across four interpersonal dimensions (warm, competent, likeable, and successful; 0 = not at all, 100 = extremely), and reported their perception of their target's self‐esteem (0 = extremely low, 100 = extremely high). Lastly, participants indicated their perceptions of their target's political orientation (0 = extremely liberal, 100 = extremely conservative).

#### 9.2.4 Narcissistic Personality Inventory—Likert Version

Participants completed the Likert rating NPI (Gentile et al. 2013). Our university participants completed the NPI‐40; for reasons of economy, our Prolific participants completed the NPI‐13. For all participants, an NPI score was derived based on the NPI‐13 items (*M* = 3.18; SD = 0.94; both *α* > 0.87).

#### 9.2.5 Participant Personality Traits

Next, participants completed the Ten‐Item Personality Inventory (TIPI; Gosling et al. 2003; “I see myself as anxious, easily upset”; 1 = strongly disagree, 7 = strongly agree). Following this, participants completed two one‐item measures of self‐esteem. As an indirect measure of self‐esteem, participants completed the Name‐Liking measure (Gebauer et al. 2008), whereby they indicated how much they liked their own name (1 = not at all, 7 = very much). As a direct measure of self‐esteem, participants completed the Single‐Item Self‐Esteem Scale (Robins et al. 2001), by indicating their agreement with the statement: “I have high self‐esteem” (1 = not very true of me, 7 = very true of me). All of these measures have acceptable psychometric properties.

#### 9.2.6 Familiarity and Confidence

Participants then reported their familiarity with, and confidence in, their understanding of their assigned term (e.g., “narcissism” or “selflessness”) using a slider scale (0 = Not at all familiar/confident, 100 = extremely familiar/confident). As familiarity and confidence scores strongly positively correlated in both the narcissistic (*r*(317) = 0.80, *p* < 0.001) and selfless (*r*(323) = 0.73, *p* < 0.001) conditions, we computed an index of the two scores labeled “knowledge”.

Following this, we asked participants to define their assigned term. Participants were told that they could provide as much detail as they wished and reminded not to use any tools to assist them. There was no time limit given for the task. The percentages of narcissism definitions receiving each Narrative Narcissism Code from Study 2 can be found in Table S3. Lastly, participants reported their age, gender, and political orientation (0 = extremely liberal, 100 = extremely conservative).

## 10 Results

We start by describing participants' self‐reported knowledge about narcissism and selflessness, before highlighting the most frequently listed words used to describe narcissistic and selfless acquaintances. Next, we report the effects of participant narcissism, experimental condition (i.e., allocation to the narcissistic or selfless condition) and their interaction on perceptions of acquaintances' desirability, values, favorability, and Big 5. As they were exploratory, findings on self‐esteem and political orientation are reported in Supporting Information (see Table S4).

### 10.1 Self‐Reported Knowledge

Participants reported significantly less knowledge of “narcissism” compared to ‘selflessness’ (*M*_narc_ = 72.09, *M*_self_ = 85.91; *t*(588.88) = −10.69, *p* < 0.001, Cohen's *d* = 0.85). There was a small but significant correlation between participant NPI scores and knowledge of narcissism, *r*(317) = 0.166, *p* = 0.003.

### 10.2 Descriptions of Narcissistic and Selfless Acquaintances

The most frequently listed words used to describe narcissistic and selfless targets are shown in Table 3. Participants listed 555 words to describe narcissistic acquaintances, and 327 to describe selfless acquaintances. “Selfish” was chosen most often to describe a narcissistic acquaintance, followed by “self‐centered”, aligned with the thematic analyses in Study 1, which focused on the concept of narcissism.

**TABLE 3:** Words listed ten or more times to describe narcissistic and selfless targets (Study 2).

Narcissistic	Selfless
Selfish (170)	Kind (240)
Self‐centered (85)	Caring (165)
Rude (65)	Generous (153)
Arrogant (63)	Thoughtful (78)
Manipulative (63)	Loving (71)
Vain (54)	Giving (64)
Egotistical (34)	Helpful (52)
Self‐absorbed (33)	Empathetic (41)
Mean (29)	Considerate (37)
Self‐obsessed (25)	Compassionate (37)
Controlling (23)	Friendly (35)
Confident (21)	Humble (21)
Annoying (21)	Happy (20)
Fake (16)	Reliable (20)
Cold (16)	Nice (18)
Ignorant (16)	Warm (16)
Entitled (17)	Understanding (15)
Angry (15)	Supportive (15)
Attention‐seeking (13)	Charitable (14)
Obsessed (14)	Honest (14)
Cocky (14)	Sweet (14)
Uncaring (13)	Patient (11)
Loud (11)	Forgiving (11)
Nasty (11)	Loyal (10)

### 10.3 Perceptions of Narcissistic and Selfless Acquaintances

First, we conducted analyses of absolute differences on mean ratings (i.e., the degree to which narcissistic and selfless acquaintances were perceived as actively evincing a specific attribute, value, etc.; see Table 4). This was achieved via one‐sample t‐tests on differences between participants' ratings on each outcome and the scale midpoint.^1^ Narcissistic acquaintances were seen as significantly less endorsing of self‐transcendence and conservation values and significantly more endorsing of self‐enhancing values than the scale midpoint. Furthermore, in line with the trifurcated model of narcissism, narcissistic individuals were seen as significantly less agreeable and conscientious, and significantly more extraverted and neurotic relative to the scale midpoint. Narcissistic individuals were also perceived less favorably (on an index of warmth, likeability, competent and successful) relative to the scale midpoint.

**TABLE 4:** NPI, condition, and NPI × condition predictors of perception ratings (Study 2).

	Narcissistic		Selfless		Predictors	*B* (SE)	*t*	BS CI
	*M*	SD	*M*	SD				
Attributes								
Listed attributes	2.10***	0.81	6.48***	0.61	NPI**	−0.27 (0.09)	−2.93	[−0.45, −0.09]
					Cond***	−4.37 (0.06)	−77.70	[−4.48, −4.26]
					NPI × Cond**	0.19 (0.06)	3.13	[0.07, 0.31]
Favorability	40.02***	20.28	83.56***	11.81	NPI**	−5.74 (2.12)	−2.70	[−9.90, −1.57]
					Cond***	−43.53 (1.31)	−33.30	[−46.10, −40.96]
					NPI × Cond**	3.87 (1.40)	2.77	[1.13, 6.62]
Values								
Self‐transcendence	29.50***	24.39	76.02***	17.59	NPI	−5.61 (2.72)	−2.06	[−10.96, −0.27]
					Cond***	−46.35 (1.68)	−27.67	[−49.64, −43.06]
					NPI × Cond*	4.61 (1.79)	2.57	[1.09, 8.14]
Self‐enhancement	76.31***	22.86	39.00***	24.34	NPI**	8.46 (3.03)	2.80	[2.53, 14.41]
					Cond***	37.52 (1.86)	20.15	[33.86, 41.17]
					NPI × Cond*	−4.59 (1.99)	−2.30	[−8.51, −0.68]
Openness	50.66	28.51	65.33***	22.25	NPI	0.98 (3.27)	0.30	[−5.46, 7.42]
					Cond***	−14.30 (2.02)	−7.08	[−18.26, −10.33]
					NPI × Cond	1.15 (2.16)	0.53	[−3.10, 5.39]
Conservation	32.04***	24.89	62.15***	24.64	NPI	5.65 (3.18)	1.78	[−0.59, 11.89]
					Cond***	−29.77 (1.95)	−15.23	[−33.61, −25.93]
					NPI × Cond	−2.10 (2.09)	−1.00	[−6.22, 2.01]
Big 5								
Agreeableness	23.79***	15.68	77.40***	16.70	NPI***	−8.16 (2.07)	−3.95	[−12.21, −4.10]
					Cond***	−53.65 (1.27)	−42.22	[−56.15, −51.16]
					NPI × Cond***	5.23 (1.36)	3.84	[2.55, 7.90]
Conscientiousness	47.37*	23.88	75.60***	16.69	NPI	−5.28 (2.64)	−2.00	[−10.47, −0.10]
					Cond***	−28.41 (1.63)	−17.47	[−31.60, −25.21]
					NPI × Cond	2.65 (1.74)	1.52	[−0.77, 6.08]
Extraversion	67.82***	20.21	62.67***	23.43	NPI	2.61 (2.82)	0.92	[−2.94, 8.15]
					Cond**	5.12 (1.74)	2.95	[1.70, 8.53]
					NPI × Cond	−1.86 (1.86)	−1.00	[−5.52, 1.79]
Neuroticism	62.50***	21.18	35.16***	26.48	NPI	2.26 (3.09)	0.73	[−3.81, 8.33]
					Cond***	27.54 (1.90)	14.48	[23.81, 31.28]
					NPI × Cond	−0.51 (2.04)	−0.25	[−4.51, 3.50]
Openness	50.18	20.29	74.17***	23.25	NPI	1.41 (2.82)	0.50	[−4.12, 6.94]
					Cond***	−23.91 (1.73)	−13.80	[−27.31, −20.51]
					NPI × Cond	−0.55 (1.86)	−0.29	[−4.19, 3.10]

*Note:* Mean values are compared versus scale midpoint. Standard errors are given in parenthesis. Cond refers to “selfless” (0) vs. “narcissistic” (1) acquaintance experimental manipulation.

Abbreviation: NPI, narcissistic personality inventory.

* *p* < 0.05.

** *p* < 0.01.

*** *p* < 0.005.

Next, to examine the effect of condition, participant NPI, and their interaction on perceptions of acquaintances' desirability, values, Big Five traits, interpersonal traits, self‐esteem, and political orientation, we ran a series of moderated regression analyses using Hayes' (2022) PROCESS macro (95% confidence intervals based on 10,000 bootstrap samples; see Table 4). We mean‐centered predictor variables and used simple slopes analysis to estimate the effect of the independent variable (participant NPI) on perception ratings at each level of the moderator variable.^2^ Across all models, we also tested whether results were influenced by sample type (Prolific vs. student participants). Including sample type as a covariate did not alter the significance of any main or interaction effects, and the covariate itself was not significant in any analysis. Full model outputs are available on the project's OSF repository.

#### 10.3.1 Desirability of Listed Attributes

To begin, we examined the effect of condition, participant NPI, and their interaction on the perceived desirability of listed attributes (we computed an average valence rating for each participant). As expected, we found a significant main effect of condition, *b* = −4.37, SE = 0.056, *t* = −77.70, *p* = 0.003. Overall, attributes linked with narcissistic acquaintances (*M* = 2.10; SD = 0.81) were evaluated more negatively than attributes linked with selfless acquaintances (*M* = 6.48; SD = 0.61). The main effect of participant NPI was non‐significant (*b* = 0.14, SE = 0.030, *t* = 0.47, *p* = 0.642). The interaction was significant, *b* = 0.19, SE = 0.060, *t* = 3.13, *p* = 0.005 (*ΔR* ^2^ = 0.0015). NPI scores were positively linked with perceived desirability of a narcissistic acquaintance (*b* = 0.11, SE = 0.045, *t* = 2.41, *p* = 0.016) and negatively linked with perceived desirability of a selfless acquaintance (*b* = −0.080, SE = 0.040, *t* = −2.00, *p* = 0.046).

#### 10.3.2 Perceptions of Warmth, Liking, Competence, and Success

As the perceived warmth, liking, competence, and success variables demonstrated moderate to large intercorrelations in both conditions (*r*s = 0.27–0.88), we examined their underlying factor structure using exploratory factor analysis (Direct Oblimin rotation). All items loaded onto a single factor (see Table S5), so we computed a “favorability” rating for each participant comprised of their average scores on all four items. For brevity, we report the favorability index analyses. Analyses on individual items are reported in the Supporting Information (see Table S6).

As expected, the main effect of condition was significant, *b* = −43.49, SE = 1.31, *t* = −33.31, *p* = 0.003. Overall, narcissistic acquaintances (*M* = 40.02, SD = 20.28) were rated less favorably than selfless acquaintances (*M* = 83.56; SD = 11.81). The main effect of participant NPI was non‐significant (*b* = 0.04, SE = 0.70, *t* = −0.058, *p* = 0.954). The interaction was significant, *b* = 3.84, SE = 1.40, *t* = 2.74, *p* = 0.012 (Δ*R* ^2^ = 0.0043). NPI scores were positively (but not significantly) linked with favorability ratings of narcissistic acquaintances (*b* = 1.98, SE = 1.05, *t* = 1.88, *p* = 0.061) and negatively linked with favorability ratings of selfless acquaintances (*b* = −1.86, SE = 0.92, *t* = −2.02, *p* = 0.044).

#### 10.3.3 Value Importance

We examined the effect of participant NPI, experimental condition, and their interaction on Schwartz's four value types. For self‐transcendence values, we found a significant main effect of condition, *b* = −46.35, SE = 1.68, *t* = −27.67, *p* = 0.003. Overall, narcissistic acquaintances (*M* = 29.50, SD = 24.39) were judged as less likely to perceive self‐transcendence values as important relative to selfless acquaintances (*M* = 76.02; SD = 17.59). The main effect of participant NPI was non‐significant (*b* = 1.28, SE = 0.90, *t* = −1.43, *p* = 0.153). The interaction was significant, *b* = 4.61, SE = 1.79, *t* = 2.57, *p* = 0.017 (Δ*R* ^2^ = 0.0047). NPI scores were positively linked with perceiving narcissistic acquaintances as placing more importance on self‐transcendence values (*b* = 3.61, SE = 1.35, *t* = 2.67, *p* = 0.008). There was no effect of NPI scores on judgments of selfless acquaintances (*b* = −1.00, SE = 1.18, *t* = −0.85, *p* = 0.397).

For self‐enhancement values, we found a significant main effect of condition, *b* = 37.51, SE = 1.86, *t* = 20.15, *p* = 0.003. Overall, narcissistic acquaintances (*M* = 76.31, SD = 22.86) were rated as more likely to perceive self‐enhancement values as important relative to selfless acquaintances (*M* = 39.00; SD = 24.34). The main effect of participant NPI was non‐significant (*b* = 1.60, SE = 1.00, *t* = 1.60, *p* = 0.109). The interaction was significant, *b* = −4.59, SE = 2.00, *t* = −2.30, *p* = 0.036 (Δ*R* ^2^ = 0.0051). NPI scores were positively linked with perceiving selfless acquaintances as placing more importance on self‐enhancement values (*b* = 3.87, SE = 1.31, *t* = 2.95, *p* = 0.003). There was no effect of NPI scores on judgments of narcissistic acquaintances (*b* = −0.72, SE = 1.50, *t* = −0.48, *p* = 0.658).

For openness values, we found a significant main effect of condition, *b* = −14.30, SE = 2.02, *t* = −7.08, *p* = 0.003. Overall, narcissistic acquaintances (*M* = 50.66, SD = 28.51) were rated as less likely to perceive openness values as important relative to selfless acquaintances (*M* = 65.33; SD = 22.25). The main effect of NPI was significant, *b* = 2.70, SE = 1.08, *t* = 2.50, *p* = 0.013, such that higher NPI scores were associated with higher openness ratings overall. The interaction was non‐significant (*b* = 1.15, SE = 2.16, *t* = 0.53, *p* = 0.678).

For conservation values, we found a significant main effect of condition, *b* = −29.77, SE = 1.95, *t* = −15.23, *p* = 0.003. Overall, narcissistic acquaintances (*M* = 32.04, SD = 24.89) were rated as less likely to perceive conservation values as important relative to selfless acquaintances (*M* = 62.15; SD = 24.64). The main effect of NPI was significant, *b* = 2.51, SE = 1.05, *t* = 2.40, *p* = 0.017, indicating that higher NPI scores were associated with higher perceived conservation values across conditions. The interaction was non‐significant (*b* = −2.10, SE = 2.09, *t* = −1.00, *p* = 0.402).

#### 10.3.4 Perceptions of Big Five Attributes

For agreeableness, we found a significant main effect of condition, *b* = −53.65, SE = 1.27, *t* = −42.22, *p* = 0.003. Overall, narcissistic acquaintances (*M* = 23.79, SD = 15.68) were rated as less agreeable than selfless acquaintances (*M* = 77.40; SD = 16.70). The main effect of NPI was not significant, *b* = −3.42, SE = 0.68, *t* = −0.50, *p* = 0.62. The interaction was significant, *b* = 5.23, SE = 1.36, *t* = 3.84, *p* = 0.003 (Δ*R* ^2^ = 0.0060). NPI scores were positively linked with perceived agreeableness of narcissistic acquaintances (*b* = 2.30, SE = 1.02, *t* = 2.24, *p* = 0.025) and negatively linked with perceived agreeableness of selfless acquaintances (*b* = −2.93, SE = 0.90, *t* = −3.27, *p* = 0.001).

For conscientiousness, we found a significant main effect of condition, *b* = −28.41, SE = 1.63, *t* = −17.47, *p* = 0.003. Overall, narcissistic acquaintances (*M* = 47.37, SD = 23.88) were rated as less conscientious than selfless acquaintances (*M* = 75.60; SD = 16.69). The main effect of participant NPI (*b* = −1.32, SE = 0.87, *t* = −1.52, *p* = 0.130) and interaction (*b* = 2.65, SE = 1.74, *t* = 1.52, *p* = 0.176) were non‐significant.

For extraversion, we found a significant main effect of condition, *b* = 5.12, SE = 1.74, *t* = 2.95, *p* = 0.007. Overall, narcissistic acquaintances (*M* = 67.82; SD = 20.21) were rated as more extraverted than selfless acquaintances (*M* = 62.67; SD = 23.43). The main effect of participant NPI (*b* = −0.18, SE = 0.93, *t* = −0.19, *p* = 0.847) and interaction (*b* = −1.86, SE = 1.86, *t* = −1.00, *p* = 0.402) were non‐significant.

For neuroticism, we found a significant main effect of condition, *b* = 27.54, SE = 1.90, *t* = 14.48, *p* = 0.003. Overall, narcissistic acquaintances (*M* = 62.50; SD = 21.18) were rated as more neurotic than selfless acquaintances (*M* = 35.16; SD = 26.48). The main effect of participant NPI (*b* = 1.50, SE = 1.02, *t* = 1.47, *p* = 0.141) and interaction (*b* = −0.51, SE = 2.04, *t* = −0.25, *p* = 0.804) were non‐significant.

For openness, we found a significant main effect of condition, *b* = −23.91, SE = 1.73, *t* = −13.80, *p* = 0.003. Overall, narcissistic acquaintances (*M* = 50.18; SD = 20.29) were rated as less open than selfless acquaintances (*M* = 74.17; SD = 23.25). The main effect of participant NPI (*b* = 0.59, SE = 0.93, *t* = 0.64, *p* = 0.523) and interaction (*b* = −0.55, SE = 1.86, *t* = −0.29, *p* = 0.792) was non‐significant.

## 11 Discussion

Directly paralleling our thematic analysis in Study 1, the most frequently listed term to describe narcissism was selfish. This provides further evidence that the selfishness dimension of narcissism is particularly salient when laypersons are asked to consider narcissism at the concept and person levels. Overall, the valence of participants' self‐reported attributes of a narcissistic acquaintance was more negative than those of a selfless acquaintance. Similar to Study 1, we found a positive association between participant narcissism and perceived desirability of self‐reported narcissist‐relevant attributes, such that participants scoring higher in narcissism were less negative in their ascriptions of a narcissistic acquaintance. A similar pattern was observed on the favorability index.

Regarding perceptions of narcissistic (vs. selfless) acquaintances' values, narcissistic acquaintances were perceived as placing greater importance on self‐enhancement values and less importance on self‐transcendence values, relative to selfless acquaintances. These perceptions reflect the pattern of associations between narcissism and personal values examined by Nowak et al. (2022). Further, more narcissistic participants perceived a narcissistic acquaintance as placing greater importance on self‐transcendence values relative to less narcissistic participants, as well as perceiving a selfless acquaintance as placing greater importance on self‐enhancement values.

Similarly, in terms of Big Five ratings, narcissistic (vs. selfless) individuals were rated as less open and conscientious and as more neurotic, disagreeable, and extraverted. This suggests consensus between public perceptions of their narcissistic acquaintances and the narcissistic personality profile posited by the trifurcated model of narcissism (Miller et al. 2016, 2017).

## 12 General Discussion

Narcissism fosters significant public interest and cultural fascination in contemporary society. This is especially true within the landscape of social media such as YouTube and TikTok, with a plethora of online influencers sharing content claiming to help viewers diagnose themselves, or their partners, co‐workers, and family members. Yet, despite its cultural magnetism, lay conceptualizations of narcissism—and their (non‐)convergence with academic models of the construct—are not well understood. This is important, as examinations of other psychological phenomena have revealed notable differences between academic and public understandings of core constructs (Hall et al. 2019, 2021). As such, broadening our knowledge of public conceptualizations of narcissism can offer important theoretical and psychometric insights, from aiding the development of measurement scales to predicting important social outcomes. Accordingly, across two studies, we examined public definitions of narcissism at the concept level (Study 1) and perceptions of narcissistic individuals (Study 2).

Across our studies we observed many novel findings with important implications. Regarding the content of respondents' perceptions, selfishness emerged as the most frequent theme associated with narcissism and narcissistic individuals. Whilst the prominence of selfishness suggests conceptual overlap between social‐personality models and lay understandings (e.g., the trifurcated model posits antagonism, which includes low levels of altruism, as core to narcissism), it also indicates a degree of non‐overlap with the contents of widely used measures of the construct. Understanding the content of lay perceptions of narcissism has important consequences. First, divergences between layperson and academic conceptualizations of narcissism have implications regarding academics' communications with the public on this topic (e.g., via online psychology websites). For example, to the extent that lay and clinical understandings of narcissism differ, researchers publicly disseminating their findings might consider explicitly detailing the specific facets of narcissism (e.g., entitlement, exhibitionism) associated with their outcome(s) of interest, rather than broadly referring to the term. Indeed, experts often fail to value and recognize lay perceptions and knowledge when disseminating their work (Koizumi and Yamashita 2021). Second, from a psychometric perspective, narcissism measures that explicitly use the term narcissism or narcissist (e.g., “To what extent do you agree with this statement: I am a narcissist”; Konrath et al. 2014), require a strong consensual understanding of the concept, something that can be questioned given our findings. Such differences in construal can impact how respondents answer questions.^3^

At the person‐level, narcissistic acquaintances are seen as highly extraverted, disagreeable, and albeit to a lesser degree, neurotic and unconscientious (see Table 4). These findings broadly converge with Miller et al.'s (2016, 2017) trifurcated model of narcissism which posits that narcissism is a hierarchal construct comprised of three interrelated maladaptive personality facets (agentic extraversion, antagonism, and neuroticism). That lay people rated narcissistic acquaintances as lower on neuroticism relative to extraversion and disagreeableness also support previous research suggesting that people tend to emphasis the grandiose (vs. vulnerable) aspect of narcissism in their personal concepts of the trait (e.g., Carlson et al. 2012; Miller, Lynam, et al. 2018). This may be because the personality features associated with grandiose narcissism are comparably outward focused (e.g., extraversion, assertiveness, risk‐taking) relative to traits associated with vulnerable narcissism, which tend to be more internalized (e.g., shame, envy, self‐consciousness). Overall, however, narcissistic individuals are seen as possessing more negative personality traits, and as being more (less) likely to hold self‐enhancement (self‐transcendent) values. That narcissistic individuals are generally perceived unfavorably support past findings suggesting that their popularity wains significantly after first‐meeting (Carlson et al. 2012).

Further, across both studies, evaluations of narcissism and narcissistic individuals varied as a function of the participant's own self‐reported narcissism. In accordance with the narcissistic tolerance hypothesis, high (vs. low) narcissism participants perceived narcissistic attributes and acquaintances more positively. As such, we were able to replicate the effects of narcissistic tolerance using a bottom‐up methodological approach bolstering its external validity.

### 12.1 Limitations and Future Directions

Across our studies, we used UK‐based respondents. Future research could examine potential cross‐cultural differences in perceptions of narcissism. Interestingly, there is evidence that narcissism can manifest in different forms across cultures, with its grandiose form (e.g., exhibitionism, dominance) more prevalent in independent cultures, and its vulnerable form (e.g., withdrawal, hypersensitivity) more prevalent in interdependent cultures (Jauk et al. 2021).

Further, although our comparison between lay definitions of narcissism and common narcissism measures in Study 1 utilizes widely used scales (e.g., the NPI, the FFNI), future research may benefit from directly assessing (non‐)convergence between lay definitions and the content of additional scales, particularly those that capture facets of narcissism beyond grandiosity and vulnerability (e.g., antagonistic, communal). Regarding measurement, some of our measures (e.g., Big 5) used brief, though psychometrically sound, measures of these constructs. Future research could use different measures of these constructs to further enhance generalization.

While Study 2 offers novel evidence that extends our knowledge of perceptions of narcissistic individuals, it should be noted that with our bottom‐up approach it was not feasible to directly control for any potential effects of relationship type (e.g., parental, romantic, collegial) or duration. This is relevant because narcissists' likeability has been found to decrease over time, ranging from first impressions to close‐other relationships (Carlson et al. 2012; Paulhus 1998). Future research could therefore account for relationship status and length when examining acquaintance perceptions. That said, our research represents an innovative approach to understanding perceptions of narcissism at the concept and person level; broadening investigations beyond abstract vignette ratings and peer‐nomination procedures.

Finally, adopting a bottom‐up approach to study narcissism offers other unique insights. In a separate line of work, we used another bottom‐up method—reverse correlation (Dotsch and Todorov 2012)—to study how people visually represent narcissists when the selfish and vain components of narcissism are made salient, and the implications of these visual representations (Smith et al. 2025). In this work, we found that people have very different visual representations (i.e., provide distinct classification images) of selfish and vain narcissistic individuals, and that when naïve participants are shown these images, they make very different judgments about them, with the vain narcissist image judged as more agentic and attractive than the selfish narcissist image.

### 12.2 Concluding Summary

In the age of social media and social influencers, narcissism has cultivated strong public interest and usage within the cultural sphere. In seconds, anyone can access a vast array of social media posts highlighting topics such as what people think narcissism is, the types of attributes and behaviors that people think make someone a narcissist, and guides on how to detect whether or not one's romantic partner is a narcissist. As we experience what might be referred to as a cultural moment of narcissism, it is important to understand how people think about narcissism and narcissists, how these understandings overlap (or not) with social‐personality psychology definitions, and the implications of such lay understandings. In this paper, we demonstrated that, at both the construct and person level, people most strongly associate narcissism and narcissists with social selfishness and vanity. Interestingly, although consensual perceptions of narcissism and narcissists show convergence with contemporary social‐personality models of the construct, they show limited correspondence with how narcissism is commonly measured within psychology. We also found that people perceive narcissism as undesirable, as measured across a range of interpersonal dimensions and behavioral intentions; however, perceiver narcissism moderated such judgments for evaluations of abstract narcissistic attributes. And as fascination with narcissism continues to flourish, our findings suggest that the underlying theme of vain self‐absorption that fortified Ovid's Narcissus 2000 years ago is meaningfully represented in contemporary lay conceptualizations.

## Author Contributions

S.S., T.P., and G.H. contributed to conceptualization and study design. S.S. performed data collection and formal analysis. S.S. led the writing of the original draft of the manuscript. G.H. and T.P. contributed to writing – review and editing of subsequent drafts and provided supervision. All authors reviewed and approved the final version of the manuscript.

## Conflicts of Interest

The authors declare no conflicts of interest.

## Supporting information


**Data S1.** Supporting Information.

## Data Availability

All data, analysis code, and research materials are available at [https://osf.io/vwn6z/].

## Endnotes

^1^
The (Benjamini and Hochberg 1995) procedure, which controls for false discovery rates, was used to correct for multiple comparisons of means. After applying the correction, adjusted *p*‐values were derived for each analysis, with the false discovery rate controlled at a 5% threshold. All significant differences remained significant after applying the correction.

^2^
The B‐H procedure was applied on main and interaction regression effects. Adjusted p‐values are presented in the text and Table 4.

^3^
We thank a reviewer for highlighting this important point.
