# Supplementary material for: How Do People Conceptualize Narcissism and Narcissistic Individuals?
Source: J Pers. 2025 Jul 11;94(3):394–408. doi: 10.1111/jopy.70008 (PMC13163632; doi:10.1111/jopy.70008)
Supplement: Supplementary file 1 — Data S1. Supporting Information. [file JOPY-94-394-s001.docx]

Supplementary Materials

Contents

[Table S1: Narcissism Narrative Coding Manual (Study 1) 2](#_Toc199778549)

[Table S2: Facet Coding Percentages with Examples (Study 1) 5](#_Toc199778550)

[Table S3: Narcissism Narrative Code Allocation Percentages (Study 2) 7](#_Toc199778551)

[Table S4: Perceptions of Self-Esteem and Political Orientation of Narcissistic and Selfless Targets (Study 2) 8](#_Toc199778552)

[Table S5: Factor Loadings for Favorability Items (Study 2) 9](#_Toc199778553)

[Table S6: NPI, Condition and NPI × Condition Predictors of Warmth, Competence, Liking, and Success Ratings (Study 2) 10](#_Toc199778554)

# Table S1: Narcissism Narrative Coding Manual (Study 1)

*Narcissism Narrative Coding Manual*

| **Code Name** | **Sub-codes** | **Examples** |
| --- | --- | --- |
| **Social Selfishness:**    A self-centred worldview; persistently prioritising oneself above and/or at the expense of others. | Egocentrism, egoism, self-centeredness (thinks the world revolves around you), self-interest, compulsive self-referral (e.g., always relating everything back to themselves). | “When people always and only care about themselves.”    “Putting oneself first to the detriment of other people.    “They think the world revolves around them and their wants/needs”. |
| **Vanity:**    Being excessively proud of, and preoccupied with, one's own appearance, qualities and achievements.    Highly concerned with how one presents oneself to others. | Self-admiration, self-infatuation, self-love, self-absorption self-curation, self-presentation, shallowness, superficiality. Obsessed with oneself and ones looks. | “Defines someone who has an unusually deep-seated love of the self, including body image”.    “Over the top or too much self-appreciation and love”.    “They are overly conscious of their physical appearance and their mental and physical abilities.”    “Someone who is obsessed with themselves and how others see them”.    “Their relationships may be superficial, based on appearances”. |
| **Relational Grandiosity:**    Feeling superior to others. Having an inflated sense of self-importance. Believing you are better than others. | Egotistical, self-important, “full of themselves”, believing that you are better (more intelligent, kind, beautiful, successful, etc.) than everyone else. | "The belief that one is better than others, possesses superior qualities...”    “An inflated sense of self-importance”.    “Treats others as inferior”. |
| **Impaired Empathy:**    Diminished care, compassion, and concern for others. An inability to experience the thoughts and emotions of others or to see from others’ perspectives. | Insensitive, cold, uncaring. Ignore others’ feelings. Indifferent to the thoughts, emotions and opinions of others. | “Don’t take others’ feelings into consideration”.    Narcissists have little interest in others’ emotions and viewpoints”.    “Oblivious to others’ needs”.    “They don’t concern themselves with the welfare of others...”. |
| **Social Aggression:**    An exploitative interpersonal style where one uses cunning or manipulation for personal gain. Can be controlling, harsh, demeaning and disrespectful. Gaslighting (e.g., by playing the victim). Can be superficially charming in order to later exploit. | Argumentative, patronizing, belittling, controlling, disrespectful, dismissive, thoughtless, neglectful, intolerant, exploitative, dominating (e.g., ignoring people’s boundaries), passive aggressive, deceitful. | “Manipulative and would use you to their advantage”.    “...they use other people solely for the purpose of fulfilling their own selfish desires”.    “They can be charming as a means to get others to further enhance their self-worth.”    “Try to control others using gaslighting”. |
| **Attention-Seeking:**    Engaging in exhibitionist, self-promoting behaviours to gain to attention and admiration of others or assert their superiority. | Boastful, showing-off, acclaim-seeking, need for validation, approval seeking, status-seeking. | “Having the desire to be the centre of attention”.    “...narcissistic people require constant approval from others”.    “Likes to brag about their achievements”. |
| **Deservingness:**    Believing that you are innately entitled to a great deal of attention, admiration and recognition from others and that certain rules do not apply to you. | Self-entitlement, high expectations, holds others to high standards. | “They believe everyone should admire them”.    “They believe they deserve more than anyone else...”.    “...taking credit for things they took no part in”.    “Narcissists...think their perfect and expect others to see them that way too.” |
| **Stubbornness:**  A refusal to change one’s attitude or position, or to admit one’s faults or errors. Blame-shifting. | Inflexibility, lack of accountability, resistance to external feedback (when perceived as negative), thinking you are always right. | “They won’t ever change because they don’t want to”.    “They can do no wrong, everything bad they do is someone else’s fault.”    “An inability to take accountability”.    “Thinks their always right.” |
| **Obliviousness:**    Oblivious to the impact of their actions on others and/or how they are perceived by others. | No self-awareness, blinded, ignorant. | “Fail to see how their words or actions can affect others”.    “They are actually unaware of their behaviour towards others”.    “Someone who cannot see their own faults”.    “They are often sadly deluded”. |
| **Emotional Fragility:**    A tendency toward low or unstable self-esteem and ego fragility which can result in protective self-enhancing via the degrading of others and/or excessive perceptions of self-victimhood. | A compulsive need to prove one’s superiority, excessively reactive and/or defensive to real or perceived negative feedback (ego-threat), inflated confidence to protect fragile self-esteem, deflecting negative opinions of the self onto others. | “Usually linked to poor self-esteem and the need for external validation”.  “A lack of sense of humour about oneself”    “...it can also lead to an excess of self-judgement and emotional vulnerability.”    “Puts others down in order to lift themselves up”. |

# Table S2: Facet Coding Percentages with Examples (Study 1)

*Facet Code Percentages Allocated to Narcissism Definitions*

| **Measure** | **Facet** | **(%)** | **Example(s)** |
| --- | --- | --- | --- |
| NPI-7 | Superiority | 44 | “An inflated sense of self-importance”. |
|  | Exploitativeness | 17 | “…would use you to their advantage”. |
|  | Exhibitionism | 11 | “Needing attention to be on you”. |
|  | Entitlement | 10 | “Someone who believes that their needs must always come first”. |
|  | Vanity | 6 | “Obsessed with their appearance” |
|  | Authority | 4 | “Someone who likes to be in control of situations”. |
|  | Self-Sufficiency | 2 | “High-degree of self-confidence”. |
| NPI-5 | Superiority | 44 | “An overly high opinion of oneself”. |
|  | Manipulativeness | 16 | “…controls the needs of others for their own pleasure”. |
|  | Exhibitionism | 10 | “…trying to attract more attention than others”. |
|  | Vanity | 7 | “Excessive vanity”. |
|  | Leadership | 5 | “… believes everything they say goes”. |
| NPI-3 | Exploitative/Entitlement | 25 | “Self-obsessed and believes the world rotates around them”. |
|  | Grandiose Exhibitionism | 15 | “Likes to be centre of attention”. |
|  | Leadership/Authority | 5 | “Controlling over others”. |
| FFNI | Entitlement | 50 | “…only interested in themselves and how things impact them”. |
|  | Arrogance | 44 | “…they think they are superior to those around them”. |
|  | Lack of Empathy | 31 | “Lacking empathy, compassion, and understanding”. |
|  | Exploitativeness | 14 | “…always try to put others down to lift themselves up”. |
|  | Manipulativeness | 11 | “…lying, deceiving, faking emotions and feelings”. |
|  | Exhibitionism | 10 | “When a person has to be the centre of attention”. |
|  | Authoritativeness | 4 | “…has to be the leader in whatever they do”. |
|  | Indifference | 2 | “…little to no interest other people’s viewpoints”. |
|  | Acclaim-Seeking | 0 | “Has to be the best or believe themselves to be the best”. |
|  | Grandiose Fantasies | 0 |  |
|  | Thrill-Seeking | 0 |  |
| GNS | Superiority | 44 | “Someone who is full of themselves”. |
|  | Exploitativeness | 23 | “…will do anything in order to get their own way”. |
|  | Entitlement | 9 | “…think they are entitled to more than others”. |
|  | Exhibitionism | 9 | “Likes to make themselves the centre of everything”. |
|  | Vanity | 6 | “Shallow, only think of themselves and how they look”. |
|  | Authority | 4 | “Someone who likes to be in control of situations”. |
|  | Self-Sufficiency | 0 | “…do not doubt their own ability”. |

*Note.* FFNI = Five Factor Narcissism Inventory; GNS = Grandiose Narcissism Scale; NPI-7, NPI-5 and NPI-3 = Narcissistic Personality Inventory seven-, five-, and three-factor solutions, respectively. Percentages exceed 100 as some participant definitions mentioned multiple codes.

# Table S3: Narcissism Narrative Code Allocation Percentages (Study 2)

*Percentages of Participant Definitions Allocated Each Narcissism Code*

|  | Code Name | Example definition | % |
| --- | --- | --- | --- |
| 1 | Social Selfishness | *“Someone who only thinks about themselves”* | 49 |
| 2 | Vanity | *“Being vain; loving yourself”* | 38 |
| 3 | Relational Grandiosity | *“Someone who feels they are superior to others”* | 32 |
| 4 | Impaired Empathy | *“Struggling to see from others’ points of view”* | 29 |
| 5 | Social Aggression | *“Gets enjoyment from putting others down”* | 26 |
| 6 | Deservingness | *“Narcissism is characterized by self-entitlement"* | 5 |
| 7 | Attention-Seeking | *“Having the desire to be the center of attention”* | 6 |
| 8 | Emotional Fragility | *“...it comes from a place of deep-seated insecurity”* | 6 |
| 9 | Obliviousness | *“Self-obsessed but unaware”* | 2 |
| 10 | Stubbornness | *“Refuses to see flaws in their behavior”* | 2 |

*Note.* Percentages exceed 100 as some participant definitions mentioned multiple codes. *N* = 111.

# Table S4: Perceptions of Self-Esteem and Political Orientation of Narcissistic and Selfless Targets (Study 2)

***Self-Esteem***

We regressed perceived self-esteem onto condition, participant NPI, and their interaction. We found no significant effects (all *p*s ≥ .180).

***Political Orientation***

A significant main effect of condition was found, *b* = 17.65, *SE* = 1.95, *t* = 9.06, *p* < .001. Overall, narcissistic acquaintances (M = 58.12; SD = 25.76) were perceived as more conservative than selfless acquaintances (M = 40.24; SD = 23.51). The main effect of participant NPI was non-significant (*p* = .087). The interaction was significant, *b* = -4.76, *SE* = 2.09, *t* = -2.28, *p* = .023. Participants scoring high on the NPI rated their narcissistic acquaintance as being less conservative relative to participants scoring low on the NPI (*p* = .009). There was no effect of participant NPI scores on judgments of selfless acquaintances (*p* = .630).

# Table S5: Factor Loadings for Favorability Items (Study 2)

*Rotated Factor Matrix for Favorability Items*

| Attribute Item | Factor Loading |
| --- | --- |
| Factor 1 – Favorability (*α* = .89) |  |
| 1. Likeable | **.84** |
| 1. Warm | **.65** |
| 1. Competence | **.65** |
| 1. Success | **.63** |

# Table S6: NPI, Condition and NPI × Condition Predictors of Warmth, Competence, Liking, and Success Ratings (Study 2)

|  | Narcissistic | | Selfless | | Predictors | *B (SE)* | *t* | *BS CI* |  |
| --- | --- | --- | --- | --- | --- | --- | --- | --- | --- |
|  | M | SD | M | SD |  |  |  |  |  |
| Attributes |  |  |  |  |  |  |  |  |  |
| Warmth | | 21.96*** | 20.33 | 85.03*** | 16.33 | NPI | 0.38 (0.78) | 0.48 | [-1.15, 1.91] |
|  | |  |  |  |  | Cond*** | -63.02 (1.46) | -43.29 | [-65.88, -60.16] |
|  | |  |  |  |  | NPI × Cond* | 3.87 (1.56) | 2.48 | [0.81, 6.93] |
| Competence | | 49.40 | 28.14 | 83.93*** | 14.03 | NPI | –0.13 (0.94) | –0.14 | [-1.98, 1.71] |
|  | |  |  |  |  | Cond*** | –34.54 (1.76) | –19.64 | [-37.99, –31.09] |
|  | |  |  |  |  | NPI × Cond | 2.31 (1.88) | 1.23 | [–1.39, 6.01] |
| Liking | | 35.21*** | 27.02 | 89.35*** | 12.78 | NPI | 0.83 (0.89) | 0.94 | [–0.91, 2.57] |
|  | |  |  |  |  | Cond*** | –54.03 (1.66) | –32.58 | [-57.28, –50.77] |
|  | |  |  |  |  | NPI × Cond** | 5.89 (1.78) | 3.32 | [2.40, 9.38] |
| Success | | 53.65* | 28.24 | 75.93*** | 18.62 | NPI** | –0.91 (1.01) | –0.90 | [–2.90, 1.08] |
|  | |  |  |  |  | Cond*** | –22.40 (1.89) | –11.85 | [–26.11, –18.69] |
|  | |  |  |  |  | NPI × Cond** | 3.29 (2.02) | 1.63 | [–0.68, 7.27] |

*Note.* Mean values are compared versus scale midpoint. Standard errors are given in parenthesis. NPI = Narcissistic Personality Inventory. Cond refers to ‘selfless’ (0) vs. ‘narcissistic’ (1) acquaintance experimental manipulation. **p* < .05; ** *p* < .01; *** *p* <.005.
